# Influence of lithium salt-induced phase separation on thermal behaviors of poly(vinylidene fluoride)/ionic liquid gels and pore/void formation by competition with crystallization†

**DOI:** 10.1039/c8ra08514e

**Published:** 2018-12-05

**Authors:** Noboru Osaka, Yuichi Minematsu, Masatoshi Tosaka

**Affiliations:** Department of Chemistry, Faculty of Science, Okayama University of Science 1-1 Ridai-cho, Kita-ku Okayama 700-0005 Japan osaka@chem.ous.ac.jp; Institute for Chemical Research, Kyoto University Gokasho Uji Kyoto 611-0011 Japan

## Abstract

The thermal behavior of poly(vinylidene fluoride)/1-ethyl-3-methylimidazolium bis(trifluoromethylsulfonyl)amide/lithium bis(trifluoromethylsulfonyl)amide (PVDF/[C_2_mim][TFSA]/LiTFSA) gels, prepared by cooling from the hot solution, was investigated with various concentrations of LiTFSA (*C*_LiTFSA_). The peak melting temperature (*T*_m_) of the gels shifted toward higher temperatures with increased *C*_LiTFSA_. However, the thickness of lamellar crystal was found to decrease with the increase in *C*_LiTFSA_, which meant that the increase in *T*_m_ was not caused by the thickening of lamellar crystal. Furthermore, we found the appearance of domains above *T*_m_ in the high *C*_LiTFSA_ region (≥20 wt%), which was a lithium ion-rich phase caused by the phase separation. Therefore, it is considered on the basis of Nishi–Wang equation that an increase in the interaction parameter with increasing *C*_LiTFSA_ toward the phase separation increased the *T*_m_. The phase-separated domains competed with the subsequent crystallization, which resulted in the formation of micrometer-sized pores and nanometer-sized voids in the spherulites. Spectral measurements revealed that PVDF was not specifically solvated in the solution state above the crystallization temperature, while [TFSA]^−^ anion formed a complex with lithium ion irrespective of the PVDF content. These results led to the consideration that an increase in the interaction parameter might be caused by the strong interaction between lithium ion and [TFSA]^−^ anion to form the complex, which would also lower the interaction between PVDF and [TFSA]^−^ anion.

## Abstract 2

Lithium salt-induced phase separation on thermal behaviors of PVDF/ionic liquid gels and pore/void structures formation by competition with crystallization.

## Graphical abstract

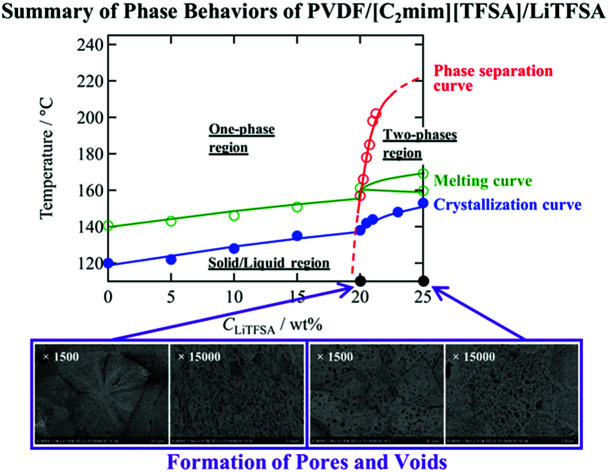

## Introduction

Ionic liquids (ILs), which are molten salts with melting points below 100 °C,^1–3^ are attracting great interest in various fields because of their high ionic conductivity and negligible volatility and inflammability. The combination of ILs with polymers is expected to further extend their applicability. Novel functions^4–7^ and physical properties^8–14^ are reported in polymer/IL systems. For example, polymer gels swollen by ILs are excellent gel polymer electrolytes (GPEs) with the properties of ILs, which includes great electrochemical stability across a large window.^15–17^ Current commercial GPEs consist of lithium salt in volatile and flammable organic solvents, such as propylene carbonate and ethylene carbonate,^18^ and accordingly, they have a potential risk of ignition. Therefore, many researchers have studied the safer IL-based GPEs to replace these flammable GPEs.

One way to fabricate the IL-based GPEs is by gelation of the polymer solution in ILs, which retains the solvents well. Polymer molecules in ILs can be cross-linked by covalent bonds^19–21^ or specific physical interactions.^22–28^ Self-assembling polymers, such as block-copolymers^29–33^ and supramolecules,^34,35^ are also used to prepare the gels. Another way to fabricate the IL-based GPEs is by impregnation of ILs into the polymer membranes, which results in better mechanical properties. For the preparation of polymer membranes in ILs, several processes, such as solvent casting,^36–40^ phase inversion,^41,42^ immersing,^43^ and electrospinning,^44–46^ were developed. However, among the above-mentioned strategies to prepare the polymer gels in ILs, the simplest way is by cooling the hot solutions of thermoreversible homopolymers in ILs, leading to the formation of crystallites as the physical cross-linking points. This simple process is not perturbed by, *e.g.*, evaporation.

As a polymer for the GPEs, copolymers of poly(vinylidene fluoride) (PVDF) are commonly used because PVDF has good chemical resistance and high dielectric constant (*ε* = 8.4), which facilitates the ionization of lithium salt. The homopolymer of PVDF is semi-crystalline, and its solution in ILs forms a physical gel on cooling due to partial crystallization. Therefore, it is expected that the essential properties of the PVDF/IL gels could be straightforwardly understood by studying the thus-formed physical gels.

It was reported that a sufficient amount of ILs promoted the formation of β phase or γ phase crystals of PVDF *via* hydrogen bonds between the C–F of PVDF and the C–H of imidazole ring of the cation,^37,40,47^ while the crystallization rate was suppressed with an increase in IL concentration.^38^ Incorporation of ILs decreased the melting point and the crystallinity of PVDF.^37,40,47^ On the other hand, the addition of lithium salt into the PVDF/ILs increased the melting point while decreasing the crystallinity. Size of the spherulites decreased with the increase in concentrations of ILs and lithium salt, and the membrane became amorphous.^40^ Despite these studies, few studies are reported on the PVDF/IL gels prepared by cooling from the hot solutions.^48^

In this study, as a model system, PVDF/IL gels containing lithium salt were prepared by dissolving PVDF and lithium salt in IL at high temperatures and subsequently cooling them. Furthermore, their thermal behavior was investigated. As the ionic liquid and lithium salt, 1-ethyl-3-methylimidazolium bis(trifluoromethylsulfonyl)amide and lithium bis(trifluoromethylsulfonyl)amide were selected, respectively, to simplify the system since they have a common anion species. We found that the addition of sufficient amount of lithium salt induced phase separation in the polymer/IL/lithium salt systems. The effect of lithium salt on the thermal behavior was discussed based on this finding. We also found that the phase separation competed with the subsequent crystallization, which led to the formation of micrometer-sized pores and nanometer-sized voids in the spherulites. Control over these specific structures would help improve the transportation of lithium ion for GPEs. Microscopic observations, wide- and small-angle X-ray scattering measurements and Fourier-transform infrared spectroscopy measurements were performed to investigate the thermal behavior from the viewpoints of hierarchical structures.

## Experimental

PVDF (*M*_w_ = 2.2 × 10^5^, *M*_w_/*M*_n_ = 2.2, KF850) was purchased from Kureha Chemical Industry Co., Ltd. 1-Ethyl-3-methylimidazolium bis(trifluoromethylsulfonyl)amide ([C_2_mim][TFSA], 99%) was purchased from IoLiTec. Lithium bis(trifluoromethylsulfonyl)amide (LiTFSA, ≥99.0%) was purchased from Sigma-Aldrich Corp. These chemicals were used without further purification, though they were vacuum-dried overnight at 90 °C. PVDF and LiTFSA were dissolved in [C_2_mim][TFSA] in a sample tube on a hot-stirrer at 210 °C. By cooling them to room temperature, the solutions turned into the corresponding gels. These gels were cut and placed between two cover glasses with a rectangular spacer of 1 mm thickness. Then, the gels were melted at 220 °C for 3 min and transferred into a hot-stage (HS82, Mettler-Toledo Inc.) at 110 °C for sufficient time to allow gelation. It is noted that we could not dissolve the phase-separated domains in PVDF/[C_2_mim][TFSA]/LiTFSA with above 21.25 wt% LiTFSA before degradation. Nevertheless, we used the gels as a series of samples to understand the effect of phase separation. Concentration of PVDF was fixed at 30 wt% against the total amount including LiTFSA, and the concentration of LiTFSA (*C*_LiTFSA_) was changed against the total amount.

A polarized optical microscope (BX-53P, Olympus Corp.) equipped with sCMOS camera (SR-130, Wraymer Inc.) was used to monitor the crystallization and phase separation of the gels. The observation area was fixed at around the center of the sample. For *in situ* observation, the hot-stage was placed on the specimen stage of the microscope.

Differential scanning calorimetry (DSC) measurements were conducted on a Q10 (TA Instruments, Inc.) at ambient pressure condition. 1–5 mg of the gels were cut and cramped into an aluminum sample pan. DSC scans were performed at a heating rate of 10 °C min^−1^.

Wide-angle X-ray scattering (WAXS) measurements were performed at room temperature on the BL40B2 beam line of SPring-8. Wavelength of the incident X-ray was 0.100 nm. The sample to detector distance was 146 mm. A flat panel detector (C9728DK-10, Hamamatsu Photonics K. K.) with 1032 × 1032 pixels (pixel size: 50 μm) was used and the exposure time was set at 10 s. The scattering intensities were corrected with respect to the background and the transmittance intensities.

Small-angle X-ray scattering (SAXS) measurements were performed using a Nano-Viewer (Rigaku Corp.). Wavelength of the incident X-ray was 0.154 nm. The sample to detector distance was 1430 mm. A two-dimensional detector (PILATUS 100K, DECTRIS Ltd.) with 346 × 487 pixels (pixel size: 172 μm) was used, and the exposure time was set at 1200 s. The scattering intensities were corrected with respect to the background and the transmittance intensities.

For Fourier-transform infrared spectroscopy (FT-IR) measurements at high temperature, Nicolet is50 (Thermo Fisher Scientific Inc.) equipped with temperature-controlled demountable liquid cell, TFC-M13-3 (Harrick Scientific Products, Inc.), with ZnSe windows and no spacer (path length ∼ 1 μm) was used. The temperature was set at 185 °C and controlled by ATC-024-3 (Harrick Scientific Products, Inc.) with an accuracy of 1.4 °C. The spectra were obtained using 64 scans at 4 cm^−1^ resolution.

Field-emission scanning electron microscopy (FE-SEM) observation was performed using SU8010 (Hitachi High-Technologies Corp.) with an accelerating voltage of 1.5 kV. Ionic liquids in the gels were removed by immersing the gels twice in sufficient amounts of acetone. Then, the samples were air-dried at 20 °C and further, vacuum-dried overnight at 20 °C. Finally, they were freeze-fractured using liquid nitrogen. The fractured surfaces of the gels were coated by sputtered Ag for observation using FE-SEM.

## Results and discussion

**Fig. 1:**
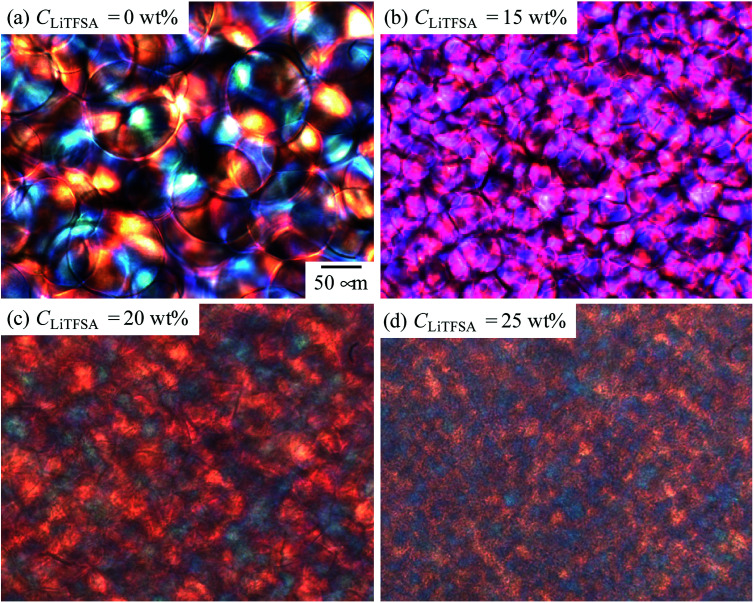
Representative polarized optical micrographs of the PVDF/[C_2_mim][TFSA]/LiTFSA gels depending on *C*_LiTFSA_.

Fig. 1 shows the representative polarized optical micrographs of PVDF/[C_2_mim][TFSA]/LiTFSA gels with various *C*_LiTFSA_. They were observed at room temperature after crystallization at 110 °C. Without LiTFSA, the PVDF/[C_2_mim][TFSA] gel showed spherulite structures with perceptible birefringence. The spherulite structures collided into each other during the crystal growth and formed a three-dimensional network structure in the gels. At 15 wt% LiTFSA, more and smaller spherulites appeared and the optical birefringence became weaker. Moreover, at or more than 20 wt% LiTFSA, the optical birefringence became stronger and mosaic-like structures appeared in the gels in addition to the spherulite structures.

**Fig. 2:**
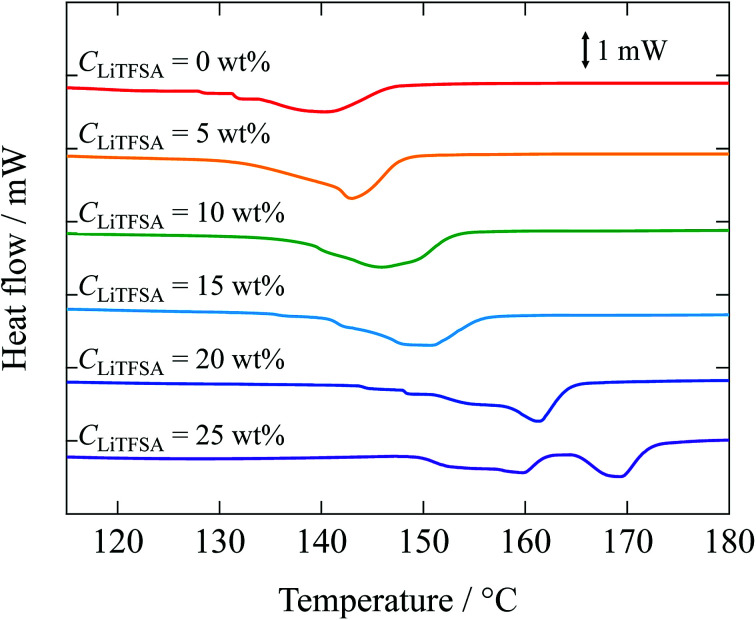
DSC curves of the PVDF/[C_2_mim][TFSA]/LiTFSA gels depending on *C*_LiTFSA_. The data are vertically offset for clarity.

Melting behaviors of the PVDF/[C_2_mim][TFSA]/LiTFSA gels with various *C*_LiTFSA_ were investigated using DSC (Fig. 2). Without LiTFSA, a broad endothermic peak appeared at around 140 °C. The peak melting temperature (*T*_m_) of the gel without LiTFSA was significantly lower than that of neat PVDF (*ca.* 170 °C).^49,50^ The depression in *T*_m_ was ascribed to the large change in the mixing entropy with the solvent addition, as described elsewhere.^51–53^ With increase in *C*_LiTFSA_, *T*_m_ shifted toward higher temperatures. Interestingly, the shift in the peak melting temperature toward higher temperature suddenly increased at around 20 wt% LiTFSA. At 25 wt% LiTFSA, double melting peaks were observed, in which one was subtly lower than that at 20 wt% LiTFSA and the other was close to that of neat PVDF. The crystallinity of PVDF (*X*_c_(DSC)) was evaluated according to the equation,1
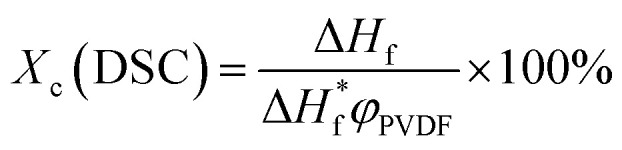
where Δ*H*_f_ and 
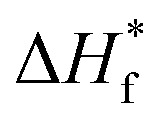
 are the enthalpies of fusion of the PVDF/[C_2_mim][TFSA]/LiTFSA gels and 100% pure crystals of PVDF (104.5 J g^−1^), respectively. *φ*_PVDF_ is the weight fraction of PVDF (30 wt%) in the gels. The above thermal parameters are listed in Table 1. While *X*_c_(DSC) was not influenced by *C*_LiTFSA_ below 20 wt% LiTFSA, *X*_c_(DSC) increased at or more than 20 wt% LiTFSA.

**Table 1:** Thermal properties of the PVDF/[C_2_mim][TFSA]/LiTFSA gels depending on *C*_LiTFSA_ obtained by DSC measurements

*C*_LiTFSA_/wt%	*T*_m_/°C	Δ*H*_m_/J g^−1^	*X*_c_(DSC)/%
0	140.6	16.5	52.7
5	143.0	16.9	53.9
10	146.0	16.6	53.0
15	150.8	16.5	52.7
20	161.2	17.4	55.5
25	159.7, 169.2	17.8	56.7

**Fig. 3:**
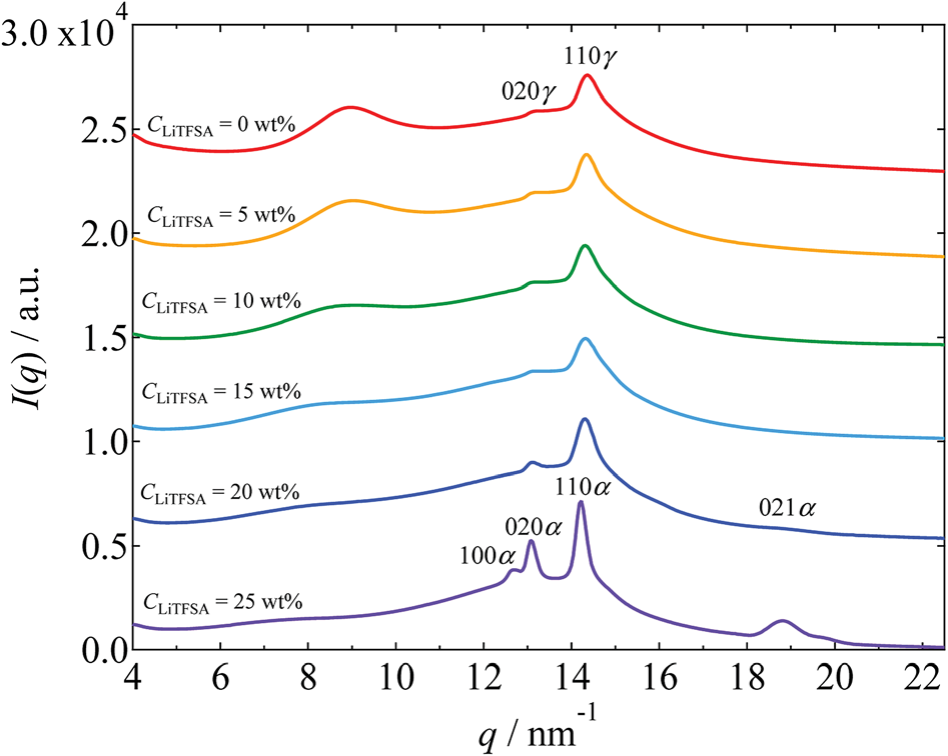
WAXS profiles of the PVDF/[C_2_mim][TFSA]/LiTFSA gels depending on *C*_LiTFSA_. The data are vertically offset for clarity.

Crystalline phase of PVDF in the PVDF/[C_2_mim][TFSA]/LiTFSA gels with various *C*_LiTFSA_ was confirmed by WAXS measurements. The WAXS profiles obtained at room temperature are shown in Fig. 3. Below 20 wt% LiTFSA, intense diffraction peaks were observed at around 14.35 nm^−1^. Also, small diffraction peaks were observed at around 13.25 nm^−1^. (The peak positions were confirmed by peak deconvolution as shown in Fig. S1.†) These peaks are attributed to the 110 and 020 reflections of the γ form, respectively.^54^ At 20 wt% LiTFSA, a subtle additional peak was observed at around 18.71 nm^−1^. This peak is attributed to the 021 reflection of the α form of the PVDF crystal. Therefore, at 20 wt% LiTFSA, the crystalline phases of PVDF consisted of majority γ form and minority α form. At 25 wt% LiTFSA, the diffraction peaks became sharper and shifted toward lower *q* values with an additional peak at around 12.68 nm^−1^. In descending order of intensity, the peaks were located at 14.22 nm^−1^, 13.09 nm^−1^, and 12.68 nm^−1^. These peaks are attributed to the α form of PVDF crystal,^54^ and they are ascribed to 110α, 020α, and 100α peaks, respectively. Thus, the crystalline phase of PVDF in the PVDF/[C_2_mim][TFSA]/LiTFSA gels was found to change from the γ form to the α form at or more than 20 wt% LiTFSA.

**Fig. 4:**
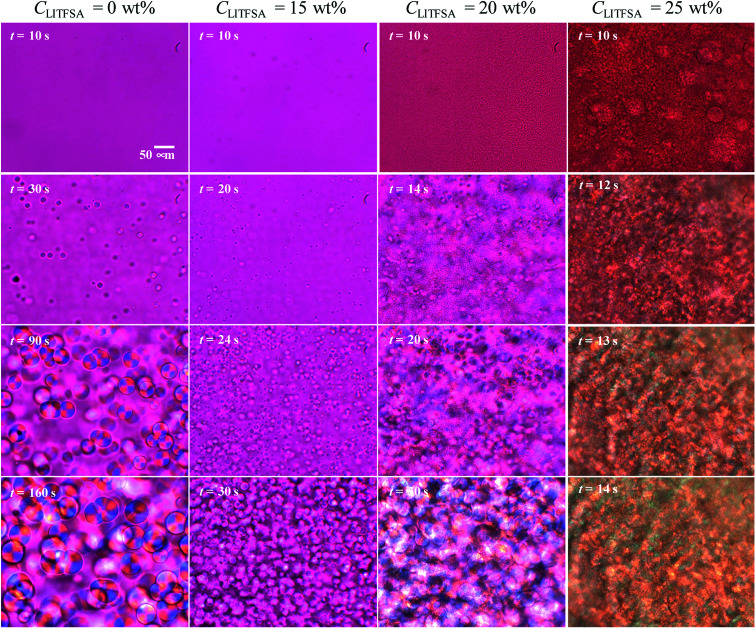
Representative timed images of the spherulite growth in the PVDF/[C_2_mim][TFSA]/LiTFSA gels at various *C*_LiTFSA_ obtained by polarized optical microscopes at 110 °C.

In order to understand the thermal behaviors of the PVDF/[C_2_mim][TFSA]/LiTFSA gels dependent on *C*_LiTFSA_, structural development of the spherulites and the nano-ordered lamellar structures of PVDF were investigated. Fig. 4 shows the representative growth behaviors of the spherulites at 110 °C as observed by the polarized optical microscope. With an increase in *C*_LiTFSA_, the emergence of spherulites started earlier and more spherulites were formed. At or more than 20 wt% LiTFSA, domains with sizes of several micrometers appeared before the onset of crystallization. Thus, it was found that more than 20 wt% LiTFSA induced phase separation in the PVDF/[C_2_mim][TFSA]/LiTFSA solutions at above the crystallization temperature. It should be noted that we could not achieve complete phase dissolution before phase degradation above 21.25 wt% LiTFSA by heating at 220 °C. This incomplete phase dissolution led to the observation of large domains with sizes of 50 μm at 25 wt% LiTFSA, as shown in Fig. 4.

Polymorphism of PVDF crystals has been well studied. The α form of PVDF crystals is the most thermally stable form and it is primarily formed at moderate conditions.^55,56^ At equal to or more than 20 wt% LiTFSA, the PVDF/[C_2_mim][TFSA]/LiTFSA solutions underwent phase separation before crystallization. Therefore, it is considered that PVDF crystallizing in the concentrated region formed the α form, as in case of neat PVDF, while PVDF formed the γ form by the interaction between PVDF and [C_2_mim][TFSA]^53^ in the low *C*_LiTFSA_ region. Thermal properties of PVDF crystals in relation to the polymorphism have also been studied.^54^ The γ form of PVDF crystals shows a higher melting temperature than the α form by about 8 °C. Therefore, the large increase in *T*_m_ at or more than 20 wt% LiTFSA cannot be ascribed to the polymorphic transition of PVDF from the γ form to the α form. The detailed mechanism is discussed in the following section based on the nano-ordered lamellar structure of PVDF and thermodynamics.

**Fig. 5:**
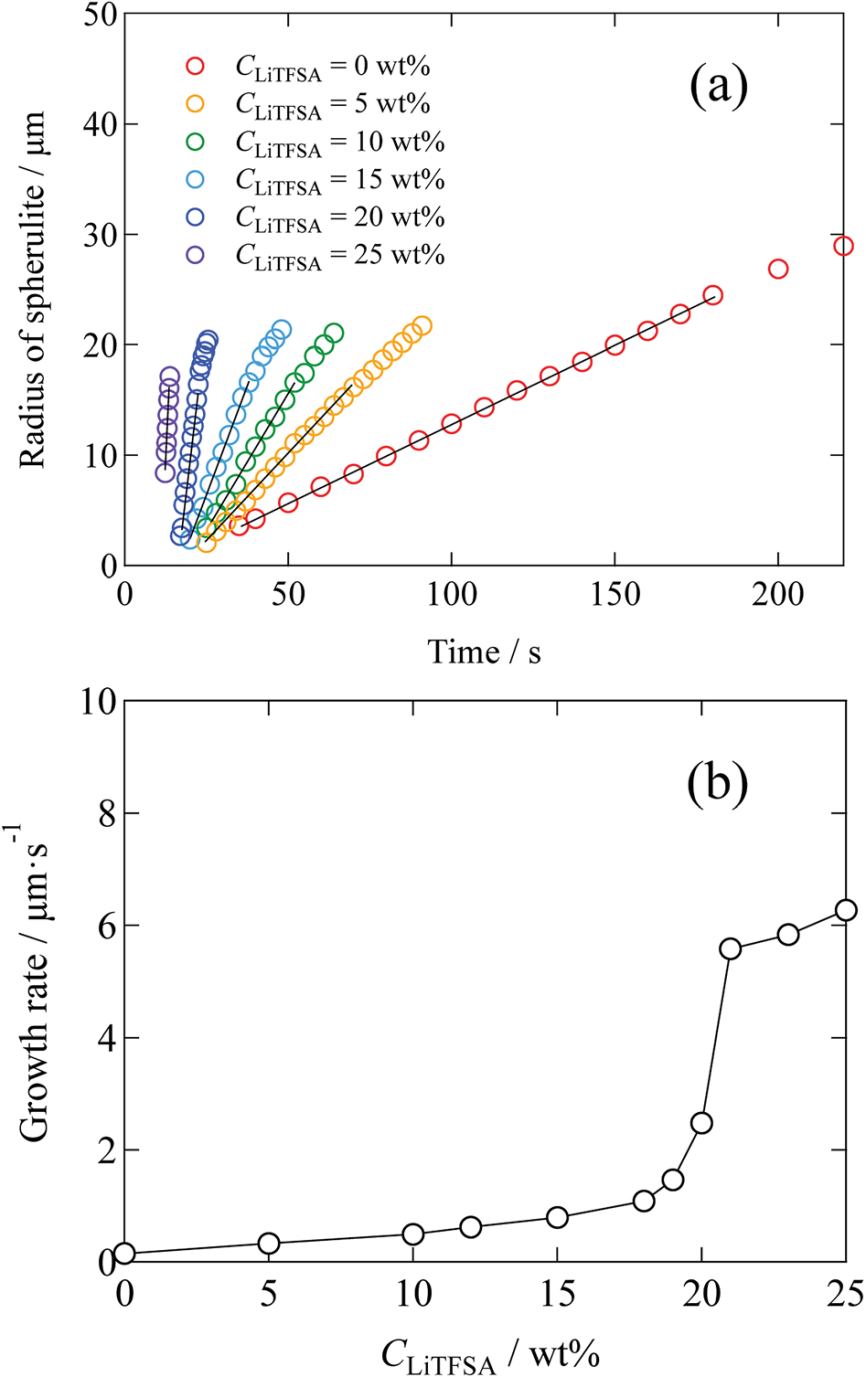
(a) Time development plots of the radii of spherulites in the PVDF/[C_2_mim][TFSA]/LiTFSA gels depending on *C*_LiTFSA_ at 110 °C. Solid lines are the linear fits to estimate the growth rates. (b) Growth rates of the spherulites depending on *C*_LiTFSA_.

Radius of the spherulites as a function of crystallization time is shown in Fig. 5(a), which was estimated from the polarized optical microscope images. Error in the radius was less than 11%. In the initial stage, since each spherulite was isolated from each other and not influenced by the surrounding counterparts, their sizes increased linearly. The growth rates of the spherulites were estimated from the linear slopes of radius *vs.* crystallization time plots. Fig. 5(b) shows the relationship between the growth rate and *C*_LiTFSA_. In the low *C*_LiTFSA_ region, the growth rate changed almost linearly with *C*_LiTFSA_. However, the growth rate suddenly increased near 20 wt% LiTFSA. This result could originate from the enhanced crystallization in the concentrated region of PVDF by phase separation, as depicted in Fig. 4.

**Fig. 6:**
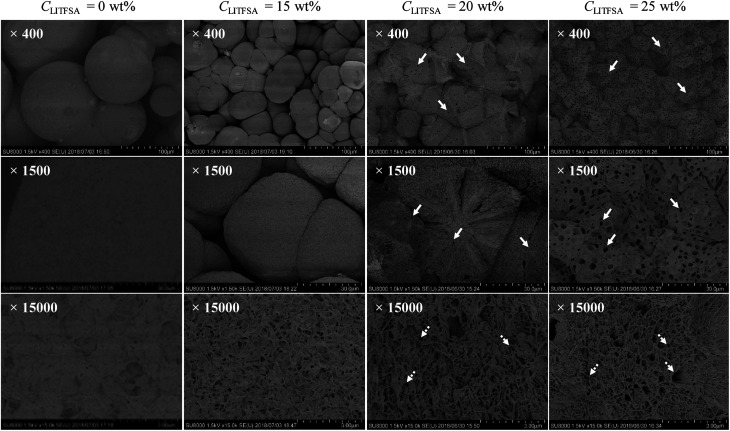
The representative FE-SEM images of the PVDF/[C_2_mim][TFSA]/LiTFSA gels depending on *C*_LiTFSA_ at various magnifications. The while solid and the white dotted arrows indicate the micrometer-sized pores and the nanometer-sized voids, respectively.

The effect of LiTFSA and the resultant phase separation in the spherulite morphology was revealed in detail by FE-SEM observations. Fig. 6 shows the representative FE-SEM images of the PVDF/[C_2_mim][TFSA]/LiTFSA gels with various *C*_LiTFSA_. With an increase in *C*_LiTFSA_, the spherulites became smaller and polygonal due to the increased number density of crystal nuclei. At or more than 20 wt% LiTFSA, where the phase separation occurred, pores with sizes of about 3 μm appeared in the spherulites. This pore formation was enhanced at 25 wt% LiTFSA. Since the sizes of the pores were comparable to those of the phase-separated domains shown in Fig. 4, the pores were formed by the competition between the phase-separated domains and the subsequent crystallization. The phase-separated domains are considered to be the lithium ion-rich phase since they were removed by acetone. As a result, the growth front of the lamellar crystal did not intrude into the domains and remained as pores on the spherulites after crystallization. At the same time, on the fractured surfaces of spherulites in gels with *C*_LiTFSA_ of 20 wt%, some radial traces were recognized. These may be the traces of extrusion of some emerging domains^57^ or viscous solvents due to formation of [TFSA]^−^/lithium ion complex^58^ from the spherulite by slow diffusion during crystallization.^59^ In addition, at the highest magnification of FE-SEM, numerous voids with sizes of hundreds of nanometers could be observed between the lamellar stacks. These small voids could be the traces of viscous solvents, which were extruded from the lamellar crystal and left behind between the lamellar stacks.

**Fig. 7:**
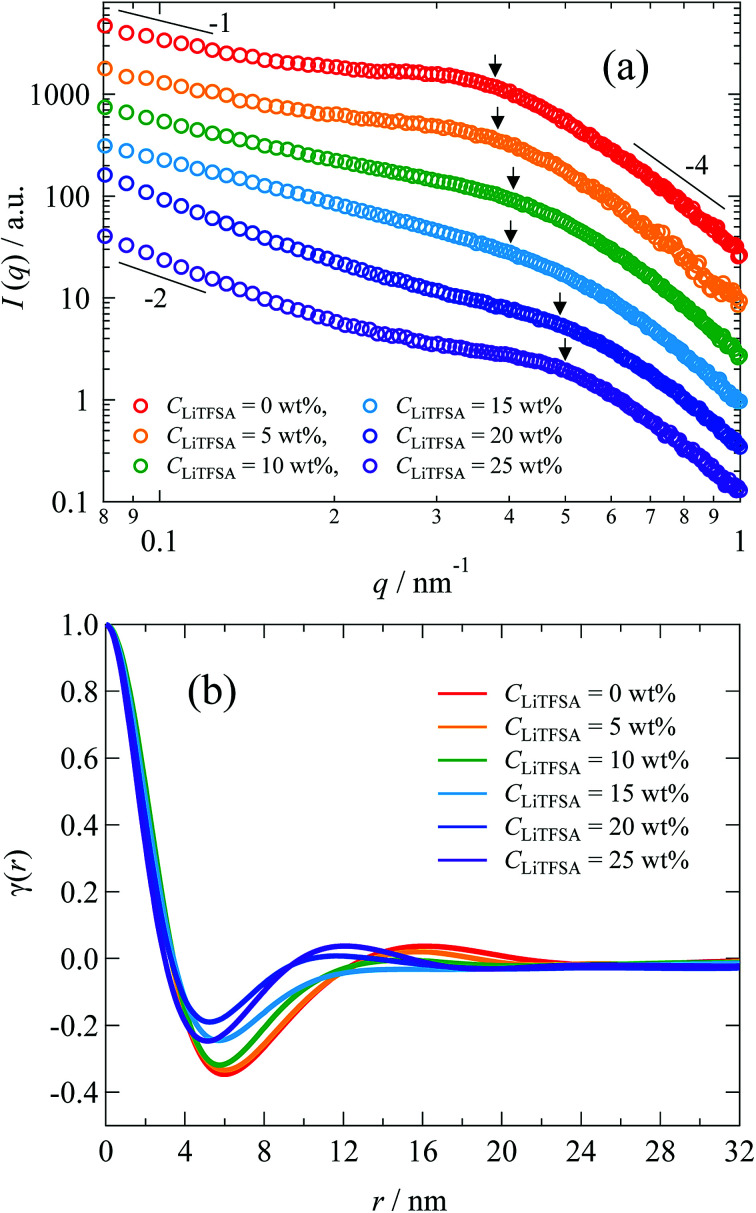
(a) SAXS curves of the PVDF/[C_2_mim][TFSA]/LiTFSA gels depending on *C*_LiTFSA_. The numbers indicate the power-law exponents. (b) Correlation functions of the PVDF/[C_2_mim][TFSA]/LiTFSA gels depending on *C*_LiTFSA_. The arrows indicate peak positions obtained from the Lorentz-corrected SAXS curves shown in Fig. S2.† The data are vertically offset for clarity.

The nano-ordered lamellar structures of PVDF were evaluated by SAXS. Fig. 7(a) shows the SAXS curves of the PVDF/[C_2_mim][TFSA]/LiTFSA gels at different *C*_LiTFSA_. Interference peaks originated from the periodic structures of lamellar crystals were observed at around 0.4 nm^−1^ in the SAXS curves. With an increase in *C*_LiTFSA_ up to 15 wt%, the peaks became unclear, which suggested a reduction in the periodic order of the lamellar crystals. Consequently, the weaker birefringence of the spherulites observed in Fig. 1 could be ascribed to this disturbance of the periodicity. In gels with or more than 20 wt% LiTFSA, the interference peaks reappeared. Crystallization of PVDF in the concentrated region by phase separation led to the reappearance of ordered periodicity of the lamellar crystal, which caused the subsequent re-entrant and larger birefringence of the spherulites. Also worth mentioning is that the power-law exponent of the scattering intensities in the low *q* region changed from −1 to −2 for gels containing equal to or more than 20 wt% LiTFSA. The power-law exponent just below the position of the interference peak represents the morphology of the lamellar crystal.^60,61^ The exponents of −1 and −2 suggest one-dimensional and two-dimensional structures, respectively. Therefore, morphologies of the lamellar crystals in the gels changed from the fibril-like structures to the plate-like structures. In the high *q* region, the power-law exponent was −4, which suggested that the interface of the lamellar crystals in the gels was smooth and sharp (Porod's law).^62,63^

To estimate the thickness of the lamellar crystal, the correlation function, *γ*(*r*), where *r* is the correlation distance, was derived by Fourier transformation on the SAXS curves according to the following equation.^63^2
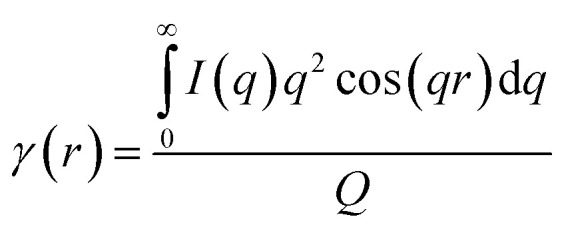
where, the scattering invariant *Q* can be expressed as3
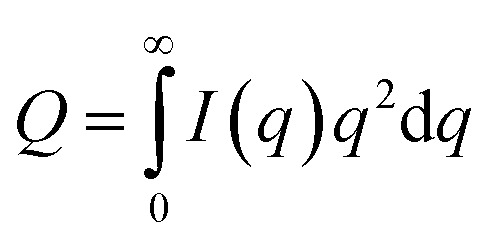


Since the data range of SAXS is inevitably limited, the SAXS curves in the low *q* region were extrapolated to *q* = 0 by fitting with the Guinier function, and the tails of the SAXS curves in the high *q* region were extrapolated to *q* = ∞ by fitting with the Sigmoid function. The obtained correlation functions are shown in Fig. 7(b). The position of the first maximum of *γ*(*r*) represents the long period of the lamellae, *L*. The *L* values were comparable to those obtained from the peak positions in the Lorentz-corrected SAXS curves *via* Bragg's equation (Fig. S2†). According to the pseudo-two-phase model, the thickness *L*_1_ was derived from the intersection between the extrapolations of the so-called self-correlation triangle and the horizontal baseline of the first minimum. The detailed procedures for the *L*_1_ calculations are described elsewhere.^62,63^ The complementary thickness, *L*_2_, was estimated as *L*_2_ = *L* − *L*_1_. When the crystallinity of PVDF is more than 50%, the thicker distance is ascribed to the thickness of lamellar crystal. In the case of our PVDF/[C_2_mim][TFSA]/LiTFSA gels, the crystallinity obtained by DSC measurements was more than 50%. Therefore, the thicker distance (*L*_2_) was attributed to the lamellar crystal (*L*_c_) and the complementary thickness (*L*_1_) was attributed to the amorphous region (*L*_a_). The obtained thicknesses by SAXS are listed in Table 2. It is noted that *L*_c_ tended to decrease with the increase in *C*_LiTFSA_.

**Table 2:** Periodic long distance (*L*), thickness of the lamellar crystal (*L*_c_) and the amorphous region (*L*_a_) of PVDF in the PVDF/[C_2_mim][TFSA]/LiTFSA gels depending on *C*_LiTFSA_ obtained by analyses of the correlation functions

*C*_LiTFSA_/wt%	*L*/nm	*L*_c_/nm	*L*_a_/nm
0	16.1	11.9	4.2
5	15.8	11.7	4.1
10	15.0	10.9	4.1
15	15.1	11.2	3.9
20	11.7	8.2	3.5
25	12.1	8.5	3.6

In a one-component system, the melting temperature of the lamellar crystal can be explained by the Gibbs–Thomson equation, which predicts that thickening of the lamellar crystal causes an increase in the melting temperature. However, our PVDF/[C_2_mim][TFSA]/LiTFSA systems are multi-component systems. Although *T*_m_ became higher with the increase in *C*_LiTFSA_, the thickness of the lamellar crystal was found to decrease. These results indicate that the increase in *T*_m_ cannot be understood within the framework of the Gibbs–Thomson equation.

Lithium ion is well known to form a complex with [TFSA]^−^ anion,^64–66^ which is the common anion between both [C_2_mim][TFSA] and LiTFSA. Therefore, we roughly assumed the PVDF/[C_2_mim][TFSA]/LiTFSA systems to be binary-component systems. Thus, the increase in *T*_m_ was discussed based on the Nishi–Wang equation,^51^ which is commonly used to understand the melting temperature depression and the interaction parameter for binary-component systems without considering the change in thickness of the lamellar crystal. In the case of polymer-solvent systems, the Nishi–Wang equation can be expressed as follows,4
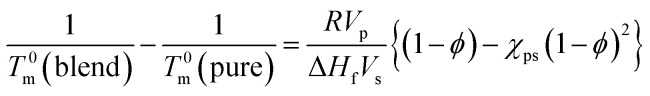
where, *T*^0^_m_(blend) and *T*^0^_m_(pure) are the equilibrium melting temperatures of the blend and the neat crystalline polymer, respectively. *R* is the gas constant and Δ*H*_f_ is the molar enthalpy of fusion. *V*_p_ and *V*_s_ are the molar volumes of polymer and solvent, respectively. *ϕ* is the volume fraction of PVDF and *χ*_ps_ is the polymer-solvent interaction parameter. Note that *χ*_ps_ actually includes contributions from the ternary interaction parameters (polymer-ionic liquid interaction parameter, polymer–lithium salt interaction parameter and ionic liquid–lithium salt interaction parameter) and the change in mixing entropy. While *R*, *V*_p_, *V*_s_, and Δ*H*_f_ are the fixed parameters, *T*^0^_m_(blend) changes with *ϕ* and/or *χ*_ps_. Change in *ϕ* either increases or decreases *T*^0^_m_(blend). Since the calculated *ϕ* in our system slightly decreased by 1 vol% and was almost unchanged regardless of the *C*_LiTFSA_, as shown in Fig. S3,†*T*_m_ can be considered to be predominantly determined by *χ*_ps_. Our PVDF/[C_2_mim][TFSA]/LiTFSA solutions underwent phase separation with an increase in *C*_LiTFSA_. In binary polymer-solvent systems, when *χ*_ps_ increases to more than 0.5, phase separation can occur. According to eqn (4), an increase in *χ*_ps_ decreases 1/*T*^0^_m_(blend), resulting in an increase in *T*^0^_m_(blend). Therefore, the increase in *T*_m_ with the increase in *C*_LiTFSA_ up to 15 wt% in our gels was ascribed to the increase in *χ*_ps_ toward the phase separation. At or more than 20 wt% LiTFSA, where the phase separation occurred before the crystallization, *L*_a_ was decreased. This result suggests that IL was considerably excluded from the gallery between the lamellar crystals of PVDF due to the segregation by phase separation. The lamellae in the concentrated region of PVDF are thought to form in solutions with less concentration of solvents, as in the case of neat PVDF. Then, the lamellar crystals with low solvent concentrations should show higher *T*_m_. At 25 wt% LiTFSA, since the dissolution temperature could not be achieved due to the degradation of crystals, the phase separation had sufficiently proceeded. In the corresponding DSC curve, which shows the double endothermic peaks, the higher *T*_m_ close to that of neat PVDF was ascribed to the melting of the lamellar crystal formed in the more concentrated region of PVDF. The lower *T*_m_ was ascribed to the melting of the lamellar crystal formed in the less concentrated region of PVDF.

**Fig. 8:**
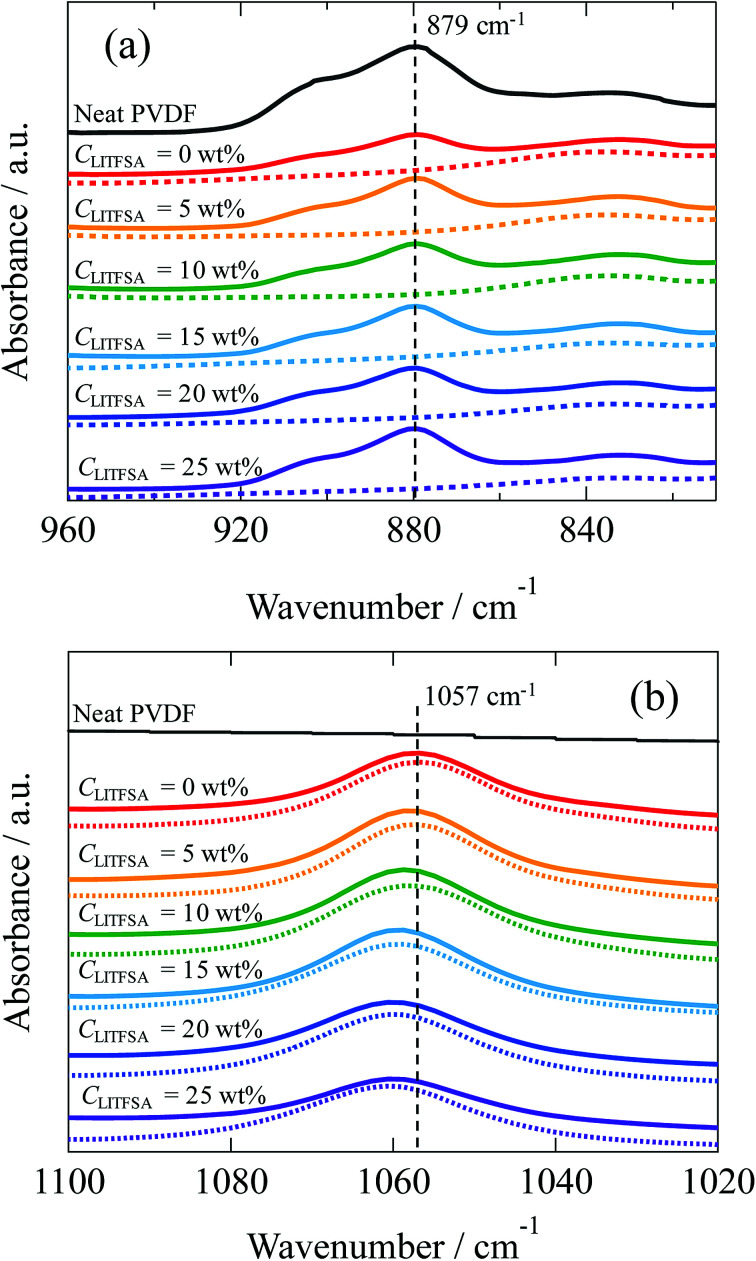
FT-IR spectra of neat PVDF (black solid curves), [C_2_mim][TFSA]/LiTFSA (dotted curves), and PVDF/[C_2_mim][TFSA]/LiTFSA solutions (solid curves) depending on *C*_LiTFSA_ obtained at 185 °C in the region of (a) the CF_2_–CH_2_ bending vibration of PVDF and (b) the symmetric stretching vibration of SO_2_ interacting with the cation. The data are vertically offset for clarity.

For further insight into the polymer-solvent interactions, FT-IR measurements were performed on the PVDF/[C_2_mim][TFSA]/LiTFSA solutions with various *C*_LiTFSA_ at 185 °C above *T*_m_. Fig. 8(a) shows the FT-IR spectra in the region of CF_2_–CH_2_ bending vibration of PVDF at 879 cm^−1^. This peak at 879 cm^−1^ was not observed in the spectrum of the [C_2_mim][TFSA]/LiTFSA solution, and accordingly, this peak in the PVDF/[C_2_mim][TFSA]/LiTFSA solutions were exclusively attributed to PVDF. Xing *et al.* reported that the peak considerably shifted toward higher wavenumber in PVDF/imidazolium-based ionic liquid with an increase in concentration of the ionic liquid at room temperature, and the obvious shift was ascribed to the specific interaction between the CF_2_ group of PVDF and the imidazolium ring of IL.^53^ However, the peak position at 879 cm^−1^ for the PVDF/[C_2_mim][TFSA]/LiTFSA solutions was neither influenced by [C_2_mim][TFSA] nor by LiTFSA (details are shown in Fig. S4(a)).† Thus, it was revealed that the CF_2_ groups of PVDF were not specifically solvated by any of the ionic species ([C_2_mim]^+^, [TFSA]^−^, and lithium ion) in the solution state at high temperatures. Fig. 8(b) shows the FT-IR spectra in the region of the symmetric stretching vibration of SO_2_ in [TFSA]^−^ anion interacting with the [C_2_mim]^+^ cation at 1057 cm^−1^.^67,68^ In the spectrum of the neat PVDF, this peak at 1057 cm^−1^ was absent, which verified that this peak was unique for the [TFSA]^−^ anion. This peak shifted toward higher wavenumber with the increase in *C*_LiTFSA_ in PVDF/[C_2_mim][TFSA]/LiTFSA solutions (details are shown in Fig. S4(b)).† Lithium ion is well known to form the complex with [TFSA]^−^ anion *via* a larger binding energy compared with that of the complex of [C_2_mim]^+^ cation and [TFSA]^−^ anion. Furthermore, lithium ion preferentially coordinates to the oxygen of the SO_2_ group of [TFSA]^−^ anion, and the resulting coordination number of lithium ion equals to two.^69^ Shift of the peaks at 1057 cm^−1^ indicated that the interaction between [C_2_mim]^+^ cation and [TFSA]^−^ anion was inhibited because of the preferential complex formation between [TFSA]^−^ anion and lithium ion, while the formation of the lithium ion complex was promoted, with an increase in *C*_LiTFSA_ solutions. The change in the peak position was almost the same between the [C_2_mim][TFSA]/LiTFSA solution and PVDF/[C_2_mim][TFSA]/LiTFSA solutions. This result indicated that the lithium ion formed a complex with the [TFSA]^−^ anion in a similar way irrespective of the presence of PVDF. Thus, non-solvating properties of the ionic species ([C_2_mim]^+^ cation, [TFSA]^−^ anion and lithium ion) toward PVDF and the strong complex formation between [TFSA]^−^ anion and lithium ion were confirmed by FT-IR measurements. From the above results, the increase in *χ*_ps_ with an increase in *C*_LiTFSA_ might be caused by the strong interaction between the lithium ion and [TFSA]^−^ anion to form the complex, which would also lower the interaction between PVDF and [TFSA]^−^ anion.

**Fig. 9:**
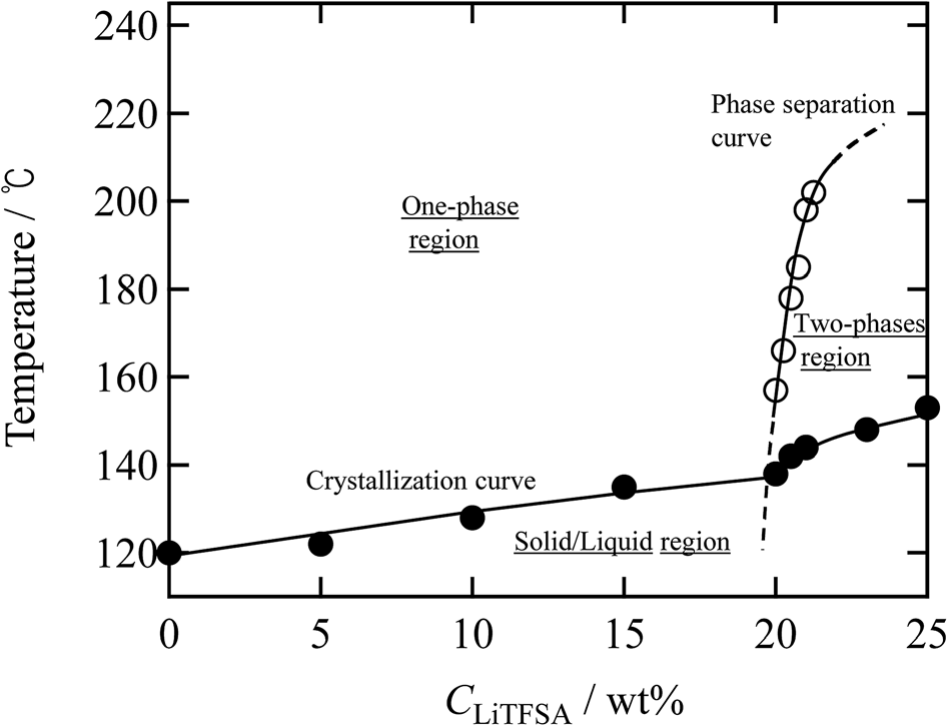
Summary of the phase behaviors of the PVDF/[C_2_mim][TFSA]/LiTFSA systems as a function of temperature and *C*_LiTFSA_ obtained by cooling at 1 °C min^−1^. The solid circles (●) represent the onset temperatures of crystallization and the opaque circles (○) represent the onset temperatures of phase separation.

Finally, a summary of the phase behaviors of the PVDF/[C_2_mim][TFSA]/LiTFSA systems was constructed based on the onset of phase separation and crystallization at various *C*_LiTFSA_ of the systems by polarized optical microscopy at a cooling rate of 1 °C min^−1^ (Fig. 9). In the low *C*_LiTFSA_ region, only the crystallization of PVDF was observed and the onset temperature increased with *C*_LiTFSA_. Since the equilibrium melting temperature increased due to the increased *χ*_ps_ toward the phase separation, the quench depth to induce crystallization became larger. The increase in the quench depth with *C*_LiTFSA_ toward the phase separation also caused the rapid growth and the crystal nucleation of the spherulites. On the other hand, at or more than 20 wt% LiTFSA, the phase separation occurred before the onset of crystallization. The onset temperature of the phase separation largely increased above 20 wt% LiTFSA. The sharp slope of the phase separation curve led to itself being buried inside the crystallization region in the low *C*_LiTFSA_ region. Therefore, we could not observe the phase separation below 20 wt% LiTFSA. In addition, the crystalline phase of PVDF consisted majorly of the γ form with a minor α form at 20 wt% LiTFSA. As shown in the summary of the phase behaviors, the onset temperature of phase separation was low at 20 wt% LiTFSA. Therefore, it is considered that the quench depth and time for the phase separation was not enough to increase the concentration of PVDF-rich phase to form the major α phase in the low *C*_LiTFSA_ region.

Fig. 4 and 6 clearly show that a sufficient amount of LiTFSA induces the phase separation in PVDF/[C_2_mim][TFSA]/LiTFSA systems. As discussed based on the results of FT-IR measurements, the phase separation was caused by the interaction between lithium ion and [TFSA]^−^ anion to form the complex, which would also lower the interaction between PVDF and [TFSA]^−^ anion. This assumption was further supported by comparing the summary of the phase behaviors of the current systems with that of the PVDF/1-ethyl-2,3-dimethylimidazolium bis(trifluoromethylsulfonyl)amide)([C_2_dmim][TFSA])/LiTFSA systems. While C2–H of the [C_2_mim]^+^ cation can have the strongest hydrogen bonding, the C2–H is methylated in the [C_2_dmim]^+^ cation. As shown in Fig. S5,† both the onset temperatures of crystallization and phase separation of the PVDF/1-ethyl-2,3-dimethylimidazolium bis(trifluoromethylsulfonyl)amide)([C_2_dmim][TFSA])/LiTFSA systems were below those of the PVDF/[C_2_mim][TFSA]/LiTFSA systems. This result suggests that PVDF in the [C_2_dmim][TFSA])/LiTFSA systems was more stable and the hydrogen bonding at C2 position of the imidazolium cation was not important for the phase transitions. However, in ternary-component systems, the origin of phase separation should be naively determined from the competition of three interaction parameters between the components (PVDF-[C_2_mim][TFSA], PVDF-LiTFSA, [C_2_mim][TFSA]-LiTFSA) in addition to the change in mixing entropy. Understanding this new phenomenon will be important for controlling the phase separation behavior and the morphology of GPEs to improve the transportation of lithium ion. Our results were obtained by preparing the gels by cooling from the respective hot solutions. However, when phase separation occurred before crystallization during an evaporation process at sufficiently high *C*_LiTFSA_, similar pore/void morphology might be obtained. The quantitative analyses will be needed in the future to establish a fundamental understanding of the thermal behaviors of the polymer/IL/lithium salt systems.

## Conclusions

The effects of lithium salt on the thermal behaviors of the PVDF/[C_2_mim][TFSA]/LiTFSA gels prepared by cooling from the hot solutions were investigated at various *C*_LiTFSA_. *T*_m_ monotonically increased below 20 wt% LiTFSA, and then suddenly increased above 20 wt% LiTFSA. Also, the addition of LiTFSA monotonically increased the growth rate of spherulites at or below 20 wt% LiTFSA and suddenly increased above 20 wt% LiTFSA. Then, the crystalline phase of PVDF transformed from the majority of γ form to the majority of α form. On the other hand, *L*_c_ was found to decrease with *C*_LiTFSA_ up to 20 wt%. This result meant that the increase in *T*_m_ was not caused by the thickening of the lamellar crystal. We found that the phase-separated domains appeared in the PVDF/[C_2_mim][TFSA]/LiTFSA solutions above *T*_m_ at or more than 20 wt% LiTFSA in gels. On the basis of the Nishi–Wang equation, it is considered that the increase in *χ*_ps_ toward the phase separation caused the increase in *T*_m_. At or more than 20 wt% LiTFSA, *L*_a_ became thinner. Because PVDF crystallized in the PVDF-rich phase, excluding the solvents, the gels consequently showed a higher *T*_m_.

Based on the spectral measurements, PVDF was found not to specifically solvate any of the ionic species in the solution state at temperatures above the *T*_m_, and the [TFSA]^−^ anion was found to form a complex with lithium ion irrespective of the PVDF concentration. These results suggested that the increase in *χ*_ps_ would be caused by the strong interaction between lithium ion and [TFSA]^−^ anion to form the complex and/or the resultant lowering of interaction between PVDF and [TFSA]^−^ anion.

As a result of the crystallization process competing with the phase-separated domains, micrometer-sized pores and nanometer-sized voids appeared in the spherulites at or more than 20 wt% LiTFSA in the gels. Some phase-separated domains were left in the spherulites as the pores. On the other hand, the extruded solvents from the lamellar crystals during crystallization would be left behind between the lamellar stacks as the voids.

## Conflicts of interest

There are no conflicts to declare.

## Supplementary Material

RA-008-C8RA08514E-s001
