# Supplementary material for: Influence of lithium salt-induced phase separation on thermal behaviors of poly(vinylidene fluoride)/ionic liquid gels and pore/void formation by competition with crystallization
Source: RSC Adv. 2018 Dec 5;8(71):40570–80. doi: 10.1039/c8ra08514e (PMC9091358; doi:10.1039/c8ra08514e)
Supplement: RA-008-C8RA08514E-s001 [file RA-008-C8RA08514E-s001.pdf]

Electronic Supplementary Material (ESI) for RSC Advances.
This journal is © The Royal Society of Chemistry 2018

Supplementary information

Influence of Lithium Salt-Induced Phase Separation on Thermal Behaviors of Poly(vinylidene fluoride)/Ionic liquid Gels and Pore/Void Formation by Competition with Crystallization

Noboru Osaka[1]*, Yuichi Minematsu[1], Masatoshi Tosaka[2]

[1]Department of Chemistry, Faculty of Science, Okayama University of Science, 1-1 Ridai-cho, Kita-ku, Okayama 700-0005, Japan

[2]Institute for Chemical Research, Kyoto University, Gokasho, Uji, Kyoto 611-0011, Japan

* Corresponding author. E-mail: osaka@chem.ous.ac.jp

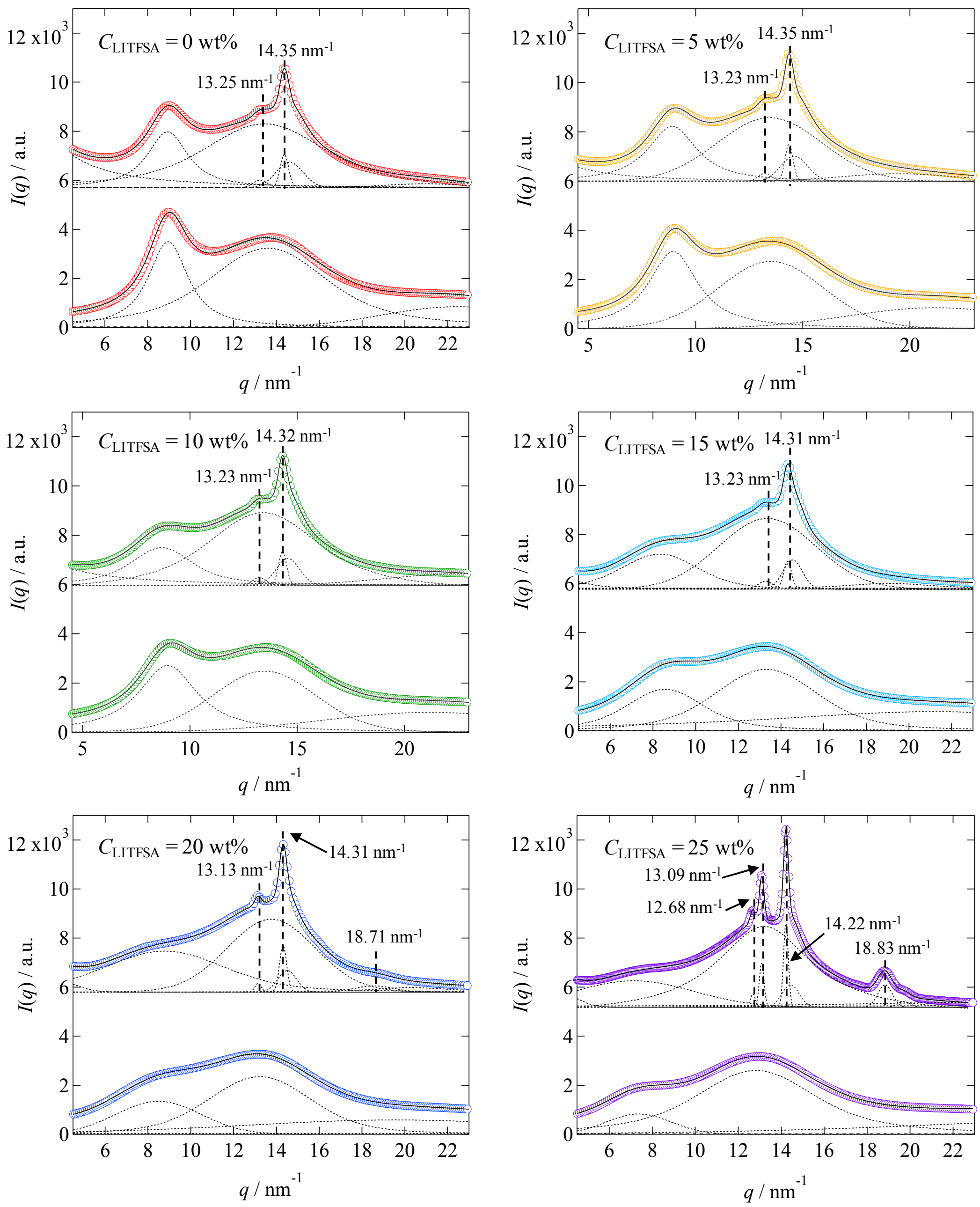

Figure S1. Results of fitting on the WAXS profiles of [C_2mim][TFSA]/LiTFSA solutions (down) and PVDF/[C_2mim][TFSA]/LiTFSA gels (top) depending on C_{LiTFSA} at 20 °C. The dotted curves for fits are Voigt functions for [C_2mim][TFSA] and Gaussian functions for PVDF, respectively.

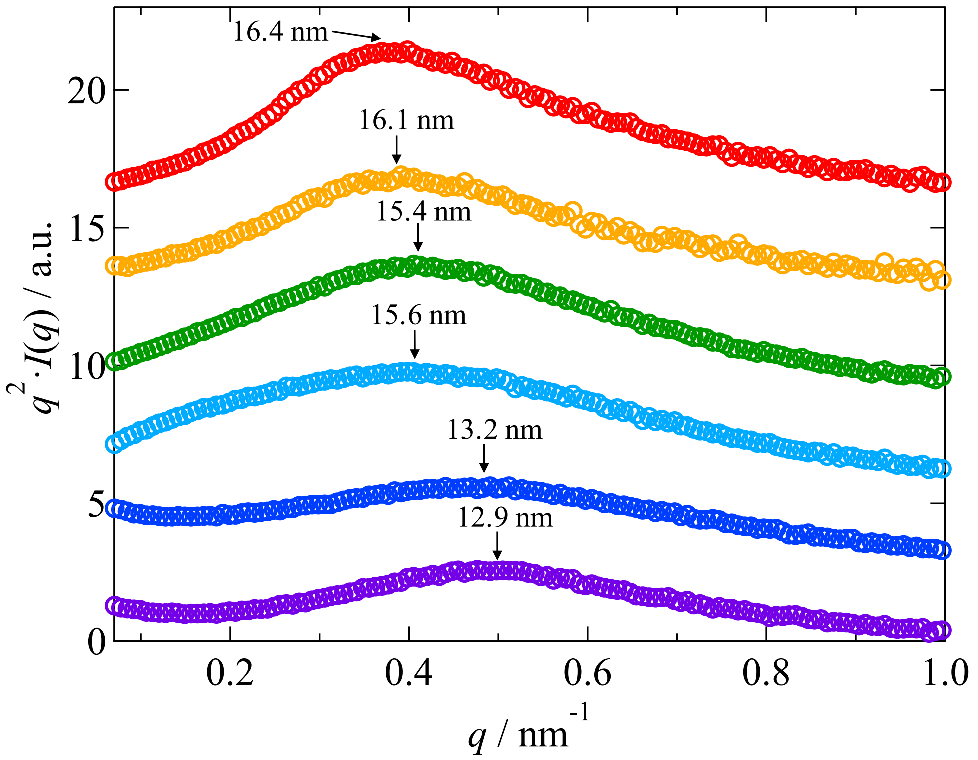

Figure S2. Lorentz-corrected SAXS curves of PVDF/[C_2mim][TFSA]/LiTFSA gels depending on C_{LiTFSA} obtained at 20 °C. The arrows indicate the peak positions and the corresponding periodic long distances obtained by Bragg's equation from the peak positions.

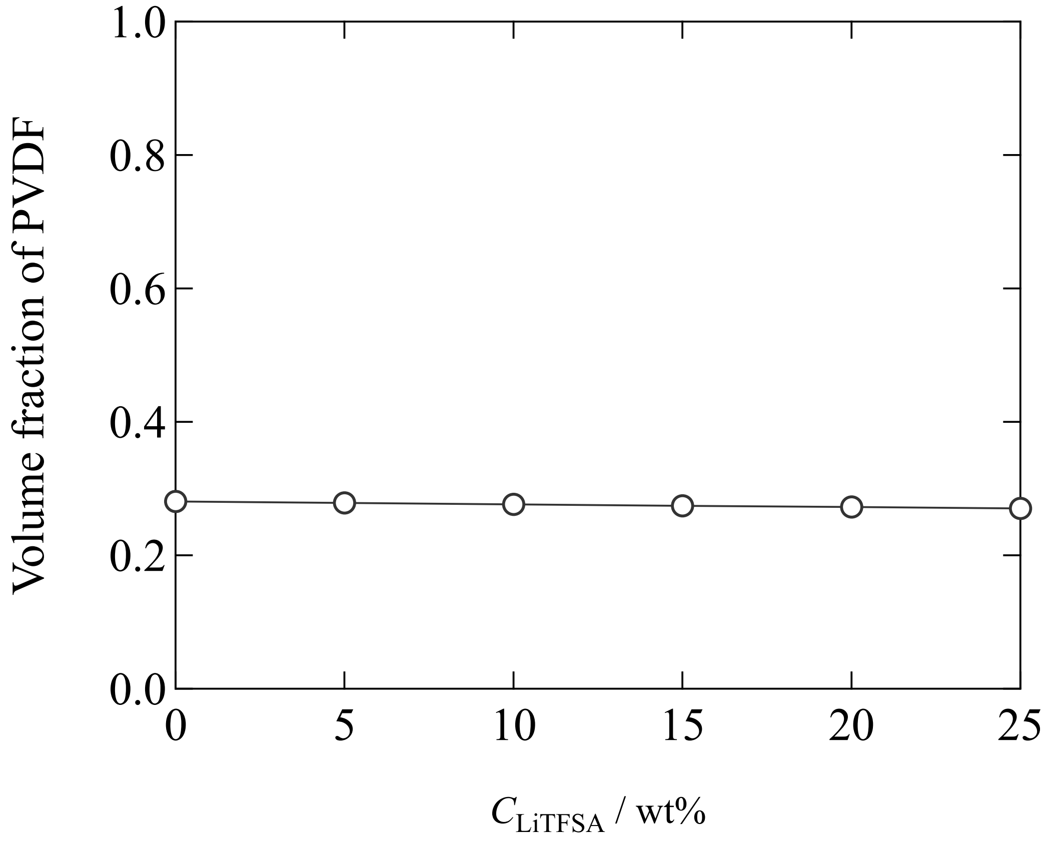

Figure S3. The estimated volume fractions of PVDF in PVDF/[C_2mim][TFSA]/LiTFSA gels depending on C_{LiTFSA}.

Volume fraction of PVDF was estimated by the following equation,

$$volume\ fraction\ of\ PVDF = \frac{\frac{\varphi_{PVDF}}{d_{PVDF}}}{\frac{\varphi_{PVDF}}{d_{PVDF}} + \frac{\varphi_{[C_2mim][TFSA]}}{d_{[C_2mim][TFSA]}} + \frac{\varphi_{LiTFSA}}{d_{LiTFSA}}}$$

φ_{PVDF}, $\varphi_{[C2mim][TFSA]}$, and φ_{LiTFSA} are weight fractions of PVDF, [C_2mim][TFSA], and LiTFSA, respectively. d_{PVDF}, $d_{[C2mim][TFSA]}$, and d_{LiTFSA} are densities of PVDF, [C_2mim][TFSA], and LiTFSA, respectively. d_{PVDF} is 1.68 g/cm^3 for amorphous PVDF [S1]. $d_{[C2mim][TFSA]}$ is 1.52 g/cm^3 for [C_2mim][TFSA] [S2]. d_{LiTFSA} is 1.33 g/cm^3 for LiTFSA [S3].

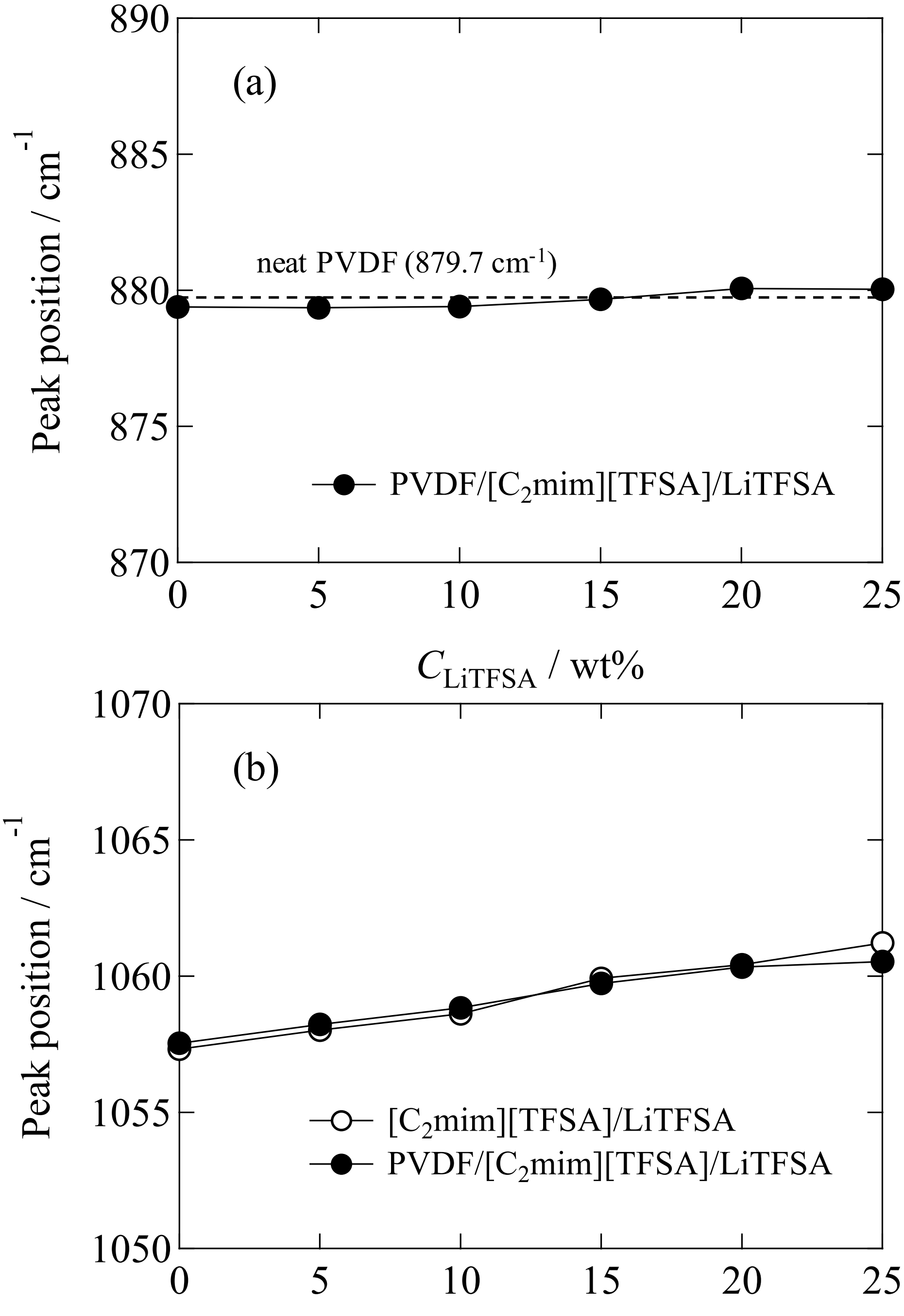

Figure S4. (a) Peak positions of FT-IR spectra depending on C_{LiTFSA} obtained at 185 °C in the regions of (a) CF_2-CH_2 bending vibration of PVDF and (b) SO_2 symmetric stretching vibration of [TFSA$^-$] anion interacting with cation.

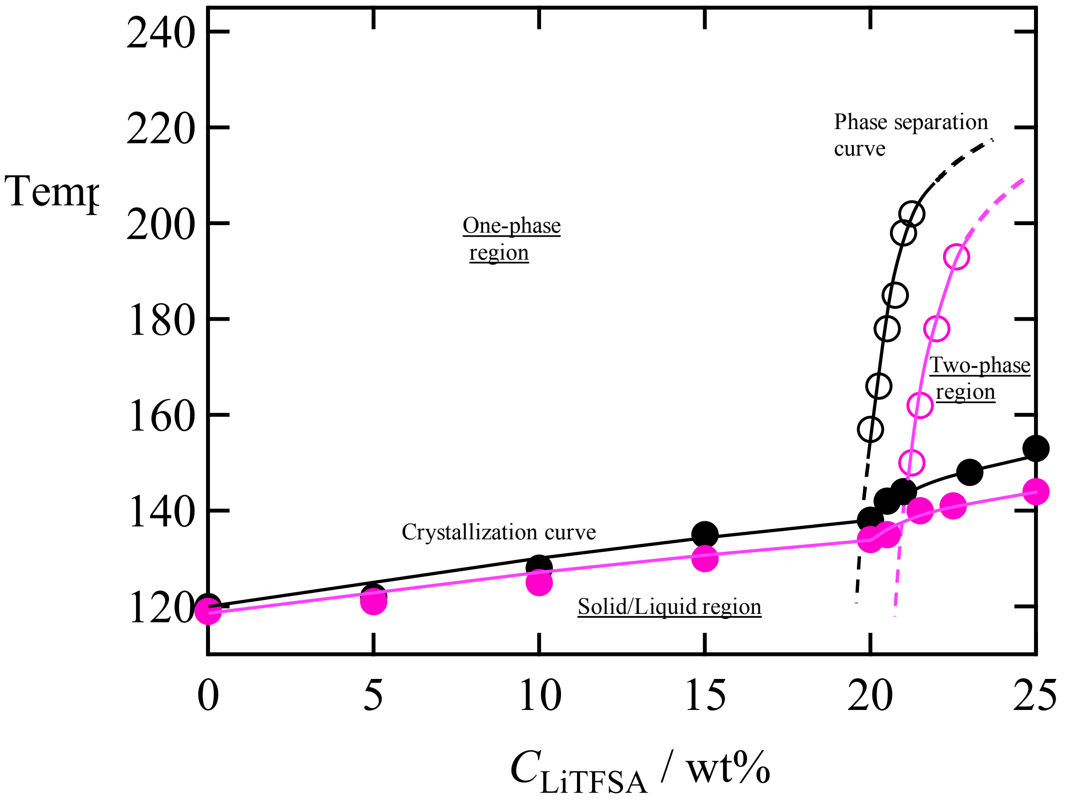

Figure S5. Summary of phase behaviors of the PVDF/[C_2mim][TFSA]/LiTFSA systems (black color) and the PVDF/[C_2dmim][TFSA]/LiTFSA systems (magenta color) obtained by cooling at 1 °C/min. Solid circles (●, ●) represent the crystallization temperatures and opaque circles (○, ○) represent the phase separation temperatures. The onset temperatures of the phase transitions were determined by using polarized optical microscope. [C_2dmim][TFSA] (99%) was purchased from IoLiTec.

References

S1. Ameduri, B., *Chem Rev,* 2009, **109**, 6632-6686.

S2. Kadyan, A.; Pandey, S., *J Chem Thermodyn*, 2018, **116**, 159-165.

S3. Lannelongue, P., Bouchal, R., Mourad, E., Bodin, C., Olarte, M., le Vot, S., Favier, F. and Fontaine, O., *J Electrochem Soc,* 2018, **165**, A657-663.
